# Nondestructive Metabolomic Fingerprinting: FTIR, NIR and Raman Spectroscopy in Food Screening

**DOI:** 10.3390/molecules28237933

**Published:** 2023-12-04

**Authors:** Nur Cebi, Hatice Bekiroglu, Azime Erarslan

**Affiliations:** 1 Food Engineering Department, Chemical-Metallurgical Faculty, Yıldız Technical University, 34210 Istanbul, Turkey; h.bkroglu@gmail.com; 2 Food Engineering Department, Faculty of Agriculture, Sirnak University, 73300 Sirnak, Turkey; 3 Bioengineering Department, Chemical-Metallurgical Faculty, Yıldız Technical University, 34210 Istanbul, Turkey; azime@yildiz.edu.tr

**Keywords:** FTIR, NIR, Raman, metabolomics, food quality, pattern recognition

## Abstract

In recent years, there has been renewed interest in the maintenance of food quality and food safety on the basis of metabolomic fingerprinting using vibrational spectroscopy combined with multivariate chemometrics. Nontargeted spectroscopy techniques such as FTIR, NIR and Raman can provide fingerprint information for metabolomic constituents in agricultural products, natural products and foods in a high-throughput, cost-effective and rapid way. In the current review, we tried to explain the capabilities of FTIR, NIR and Raman spectroscopy techniques combined with multivariate analysis for metabolic fingerprinting and profiling. Previous contributions highlighted the considerable potential of these analytical techniques for the detection and quantification of key constituents, such as aromatic amino acids, peptides, aromatic acids, carotenoids, alcohols, terpenoids and flavonoids in the food matrices. Additionally, promising results were obtained for the identification and characterization of different microorganism species such as fungus, bacterial strains and yeasts using these techniques combined with supervised and unsupervised pattern recognition techniques. In conclusion, this review summarized the cutting-edge applications of FTIR, NIR and Raman spectroscopy techniques equipped with multivariate statistics for food analysis and foodomics in the context of metabolomic fingerprinting and profiling.

## 1. Introduction

The recent term “omic” is used in a wide variety of fields, for example, genomic, proteomic, metabolomic, amongst others. The term metabolomics can be described as the cooperation of multiple disciplines such as analytical chemistry, technology, analysis protocols and data analysis [1]. Metabolomics has the capability to obtain key information about endogenous and exogenous small molecule metabolites (<1500 Da) and metabolomics can be used to evaluate a wide variety of subjects, such as toxicology, drug discovery, cancer, genetic manipulation, natural products and more [2]. These small molecules can be listed as peptides, amino acids, nucleic acids, carbohydrates, vitamins, phenolic compounds and small-molecule biomarkers [2]. Previous reports defined metabolomics as the measurement of intra- and extra-cellular metabolites in a biological system (microbial, plant and mammalian systems) [3]. A number of researchers have reported the mass spectrometry, gas chromatography–mass spectrometry (GC-MS), high-performance liquid chromatography–mass spectrometry (HPLC-MS), capillary electrophoresis–mass spectrometry (CE-MS), nuclear magnetic resonance spectroscopy and vibrational spectroscopy (infrared and Raman spectroscopy) as potential analytical techniques with various advantages and disadvantages for the measurement of the metabolome in various matrices [3]. In general, the application of a single methodology may not provide adequate information about the investigated metabolome; hence, confirmatory or additional analytical methodologies will improve the reliability and precision of the obtained results. Metabolomics can be mentioned as the most functional section among omic sciences since it has the capability to evaluate biochemical processes. Metabolomics has three cornerstones: biochemical knowledge, analytical methods and data mining chemometrics [4]. The mentioned chromatography and spectroscopy techniques may provide comprehensive metabolomics data. However, there is need for the extraction of specific metabolome-related information from big datasets using chemometrics in the untargeted analysis. In other words, there is a need for well-built multivariate statistical methods to analyze complex metabolomics data and to cope with the difficulties arising from the inherent features of datasets.

The current article was proposed to show the capability of vibrational spectroscopy techniques for metabolic fingerprinting by exploring previous contributions. This review article aims to explain the metabolic measurement properties of the vibrational spectroscopy techniques such as IR spectroscopy (MIR, NIR) and Raman spectroscopy. The classifications of infrared spectroscopy techniques and Raman spectroscopy were previously discussed in earlier contributions [3]. In the current review, we illustrate IR (MIR and NIR) and Raman spectroscopy applications for metabolomics analyses in food matrices.

## 2. Multivariate Data Analysis—Chemometrics

**Figure 1:**
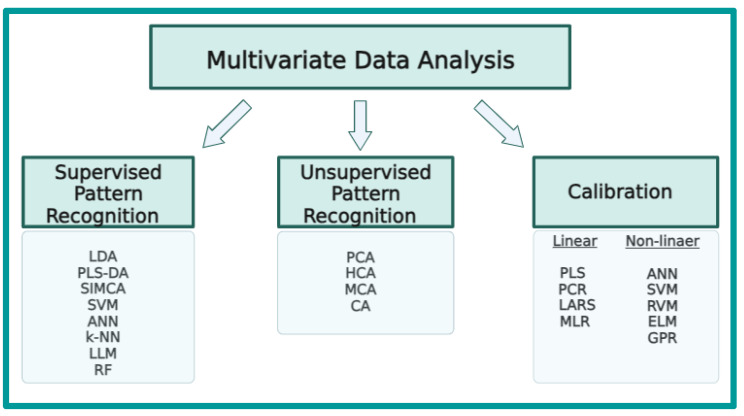
A general overview of multivariate data analysis techniques frequently employed with vibrational spectroscopy techniques.

Chemometrics can be defined as the statistical and mathematical methodologies employed to build experimental methodologies and reveal important knowledge from big data and deep data [5]. The data obtained from laboratory equipment are generally complex and require elaborated processing to obtain meaningful and critical information about the analyte. Chemometrics could be efficiently used to evaluate experimental data obtained by the laboratory equipment such as chromatography and spectroscopy systems. Chemometrics can be used to extract desired (specific) information for the classification of specific sample sets; this can be introduced as “pattern recognition” [6]. Multivariate statistics evaluates the effect of two or more independent variables on one or more dependent variables. Multivariate statistical methods may include various regression models and classification models to reveal hidden relationships between variables. From a general point of view, it is possible to state that unsupervised and supervised multivariate statistical methods have key importance for the processing of multivariate datasets. Foodomics, a novel concept, can be defined as a new discipline investigating food and nutritional issues using high-tech analytical systems and multivariate statistical approaches. Foodomics is related to lots of multidisciplinary scientific branches, such as food control and food authenticity, food processing and food functionality with the involvement of multivariate statistical analysis (chemometrics) [7]. Untargeted and targeted instrumental methods can be used to evaluate food quality and food safety issues in the food supply chain. Vibrational spectroscopy has strong capabilities for the rapid, high-throughput and nondestructive analysis of a wide variety of agricultural and natural products with powerful fingerprinting properties. Thus, vibrational spectroscopy as a fingerprinting technique can be employed for the determination of compounds in food matrices, the detection of additives and contaminants and the identification of metabolites [8]. Other advantages of vibrational spectroscopy techniques can be listed as follows: high reproducibility, in situ measurement, confirmed and validated methodologies, excellent metabolite coverage, minimum sample preparation and the direct measurement of samples in the liquid or solid state. In the chemometrics, selection of the appropriate statistical technique provides the opportunity to obtain key information about the targeted or nontargeted constituents. From a general point of view, multivariate chemometric methods can be employed with three objectives in food-related applications: (a) exploratory data analysis; (b) discrimination and classification; (c) regression and prediction. Statistical methods can be divided into two sections: unsupervised and supervised methods. Most foodomics studies include applications of pattern recognition techniques capable of determining the origin, authenticity and adulteration problems in natural and agricultural products on the basis of chemical information obtained from analytical measurements. Unsupervised pattern recognition and supervised pattern recognition are the most well-known and frequently applied techniques to cope with the challenging authenticity and fraud problems in foods, food additives, natural products and agricultural products. An overview of multivariate data analysis techniques is presented in Figure 1.

### 2.1. Unsupervised Pattern Recognition

Unsupervised pattern recognition models can be used to evaluate the connatural tendency of data without any confinements. Unsupervised methods can also be described as exploratory methods, for example, principal component analysis and hierarchical cluster analysis. These types of methodologies are usually employed to display the similarities and heterogeneities of the dataset. The most well-known and applied unsupervised chemometrics models are reported to be principal component analysis (PCA), hierarchical cluster analysis (HCA), cluster analysis (CA) and multiple correspondence analysis (MCA) [9].

### 2.2. Supervised Pattern Recognition

Supervised pattern recognition models have reported to require a definite training set, which is further used to predict the identity of unknown samples [9]. In other words, in supervised methods, spectral information is assigned to a definite class and statistical treatment is performed to display the relationship between the data and the class [10]. Supervised models can be used for classification, discrimination or prediction on the basis of analytical data produced in the laboratory. While classification models search for the similarities in the sample group on the basis of experimental information, discrimination models evaluate the divergence between the regressions of each class [9]. Both of these techniques can be used in the classification of agricultural products on the basis of their origin, growing conditions and maturity. In other words, supervised models can be used to monitor the characteristic properties of food and agricultural products in terms of process control and quality control. There are various supervised tools for the treatment of experimental data obtained from targeted or nontargeted analyses. The most well-known supervised tools are LDA (linear discriminant analysis), PLS-DA (partial least squares discriminant analysis), SIMCA (soft independent modeling of class analogy), SVM (support vector machine), RF (random forests) and ANN (artificial neural networks) [11]. Additionally, K-NN (K-nearest neighbor), LLM (linear learning machine) and Bayes linear discriminant analysis are other examples of supervised learning models [12].

### 2.3. Multivariate Calibration

The calibration section is composed of a series of subheadings, such as univariate calibration, multiple linear regression, principal component regression, partial least squares and model validation (cross-validation and independent test sets) [13]. Multivariate calibration is divided into two main components: linear and nonlinear calibration. The most well-known linear calibration methods are MLR (multiple linear regression), PLS (partial least squares regression) and PCR (principal component regression). Also, in recent years, the new linear calibration models of least angle regression (LARS) and elastic net have been introduced. Previous contributions reported the most popular nonlinear calibration models to be ANN (artificial neural network), SVM (support vector machine), RVM (relevance vector machines), ELM (extreme learning machine) and GPR (Gaussian process regression) [12]. Calibration models correlate the response and independent variables to build a statistical model that is capable of the prediction of unknown sample varieties. The fitness and validity of the models can be evaluated using some specific values such as R^2^ (regression coefficient), PRESS (prediction residual sum of squares), RPD (residual predictive deviation), SEP (standard error of prediction) and SECV (standard error of cross-validation).

### 2.4. Advantages of Pattern Recognition Techniques in Food Analysis

Food recognition, classification and discrimination are highly important tasks for food integrity and food safety from farm to fork in the food supply chain. Pattern recognition techniques can be employed for a wide variety of purposes in food-related issues. These issues include food calorie estimation, quality detection of vegetables, fruits, meat and aquatic products, the determination of food contamination and sensory evaluations [14]. Today, there is need for the application of modern statistical techniques for the quality assurance of food products in terms of the global economy. Food scientists deal with massive amounts of data produced by highly technical instrumental analysis equipment, analytical devices, sensory evaluations, and experiments, etc. [15]. The application of chemometrics as an adjunct discipline has emerged as an effective problem-solving approach in both industrial and scientific issues. Through the application of classification and calibration chemometrics models, the intrinsic relationship between variables can be revealed and, in this way, scientists can gather more knowledge and understanding in relation to the applied reference methods, the chemical composition of the samples, the applied processes and the nature of the product. Briefly, the application of pattern recognition techniques to big data gathered by different measurement systems brings advantages for the effective day-to-day analysis of foods and natural products in terms of food safety and food quality.

## 3. IR Spectroscopy Applications

### 3.1. MIR (Mid-Infrared Spectroscopy) Applications

Mid-infrared spectroscopy provides detailed information about the composition and chemical structure of the evaluated materials. An infrared spectrum of the molecule is very distinctive and presents a chemical fingerprint of the materials with characteristic functional groups [16]. FTIR (Fourier transform infrared) spectroscopy was reported to be a reliable metabolic fingerprinting technique, which is capable of high-throughput, cost-effective, easy, green analyses with minimum or no sample preparation [17]. In other words, it is possible to obtain metabolomic fingerprints for a wide variety of biofluids without any reagent or preprocessing. FTIR spectroscopy has the spectacular advantages of rapid, effective and robust detection in herbal products when compared to traditional analytical methodologies [18]. In general, the advantages of FTIR spectroscopy make the technique a good alternative for metabolic profiling and metabolic fingerprinting when compared to traditional methodologies. Metabolic profiling requires the identification and quantification of related metabolites, while metabolic fingerprinting includes the classification and screening of the analytes. An overview of reported studies using Fourier transform infrared Fourier transform infrared (FTIR), Raman and NIR spectroscopy in determination of authenticity, quality, safety and the other essential parameters in foods is presented in Table 1. In a previous contribution, Borges et al. (2014) used ATR-FTIR spectroscopy combined with PCA (principal component analysis) for the identification of distinct metabolic profiles, especially in the fingerprint regions of carbohydrates, proteins and lipids in banana accessions. According to their results, ATR-FTIR spectroscopy revealed the spectral diversity of the starchy components of banana pulp flours. In particular, the starch composition of banana accessions was favorable to distinguish accessions on the basis of their physical, chemical and functional properties [19]. Easmin et al. (2017) employed FTIR spectroscopy for the evaluation of the α-glucosidase inhibitory activity of *P. macrocarpa* extracts and an effective regression model was developed using the functional groups of -CH, -NH, -COOH and -OH for the fast and reliable determination of the inhibitory activity [20]. Their results showed that chemical groups of -CHO, -COOH, -NO_2_, -NH and -OH increased the inhibitory activity, while -SH, -PH and -PO decreased the bioactivity. Medicinal plants are important natural sources of curative and alternative treatments with health-beneficial phytochemical ingredients such as phenolics and flavonoids. FTIR spectroscopy has considerable potential for the evaluation of primary and secondary metabolites with favorable fingerprinting capabilities. Sahoo et al. (2023) successfully determined the metabolites of gallic acid, arabinogalactan protein, gingerols, shogaols, paradol, phenylalkanoids, piperine and curcumine in different herbal species, such as *Terminalia chebula*, *Zingiber officinale*, *Piper longum* and *Curcuma longa,* using metabolite-related FTIR frequencies. According to their results, FTIR analysis was successful in detecting secondary metabolites using the spectral information related to functional groups such as -CHO, -COOH, -NO_2_, -NH and -OH [21]. In a previous study, Kwon et al. (2014) evaluated the capability of FTIR spectroscopy combined with multivariate analysis for the discrimination of ginseng leaf extracts on the basis of the ages and cultivars. The results showed that the discrimination power was determined as 94.8% for cultivars and cultivation ages and the results indicated that FTIR spectroscopy combined with multivariate statistics was successful for the metabolic discrimination of cultivation ages. Significant FTIR spectral changes were correlated with variations in major metabolites (sugars and amino acids) and secondary metabolites of the ginseng leaves. Additionally, according to their results, the cultivation period was related to the metabolic variation in ginseng leaves [22]. Osman et al. (2022) evaluated the metabolome responses among wheat genotypes to heat stress using Fourier transform infrared spectroscopy. They reported that FTIR spectroscopy combined with chemometrics is a powerful technique for the characterization of the metabolic behavior of wheat genotypes under heat stress [23]. Abramovic et al. (2007) successfully employed FTIR spectroscopy for determination of deoxynivalenol in wheat [24]. In another study, Kurniawan et al. (2017) evaluated the effect of phenolic compounds and melanoidin on the antioxidant activity of Indonesia robusta and Arabica coffee extracts using FTIR spectroscopy combined with principal component analysis and regression models. They reported that the phenolic compound composition had a higher impact on the antioxidant activity when compared to melanoidin contents on the basis of principal component analysis. Additionally, partial least regression models revealed that, while hydroxyl group (O–H) contents were positively related to the antioxidant properties of the carbonyl (C=O) and amine (N–H) groups, they were negatively related to the antioxidant activity of coffee extracts [25]. Nurrulhidayah et al. (2015) used FTIR metabolite fingerprinting for the determination of lard adulteration in butter. The regression models yielded a high correlation coefficient of 0.99 and the models were successful for the determination of the lard content of adulterated samples on the basis of selected functional groups (ester/lactone, aldehyde, ketone, aromatic acids and aliphatic groups) [26]. Mycotoxins are known as toxic secondary metabolites of fungi species and a lot of food species, such as cereals, can be contaminated with them. Fu et al. (2014) used FTIR spectroscopy combined with the chemometrics of the principal component analysis for the detection of 15-acetyldeoxynivalenol (15-AcDON) in corn oil. They observed 15-AcDON-related spectral bands around the 1000 cm^−1^ wavelength; however, the detection was poor below a 1 ppm contamination level [27]. In another study, Bove et al. (2009) compared PCR and FTIR methodologies for the evaluation of biodiversity in *D. hansenii* strains isolated from pecorino cheese samples from 10 different regions of Italy. Their findings showed that the FTIR method provided more reliable results, with a 78% discrimination power for the PCoA (principal coordinate analysis algorithm), when compared to the random amplification of polymorphic DNA. The authors reported that FTIR metabolomic fingerprint clustering was weakly correlated with the observations of RAPD [28]. Skotti et al. (2014) evaluated the variations in the cellular biochemical composition of the phytopathogenic fungus *Alternaria alternate* via the effect of some selected Greek medicinal and aromatic plants using FTIR spectroscopy. The authors reported that fatty acids, amides and polysaccharides contributed to the changes in the spectral data. Additionally, it was possible to correlate fungal growth with FTIR band ratio values using spectral information. Also, the results from this study highlighted the capability of FTIR spectroscopy for the determination of variations in the main cellular components [29].

### 3.2. Raman and FT-Raman Applications

**Figure 2:**
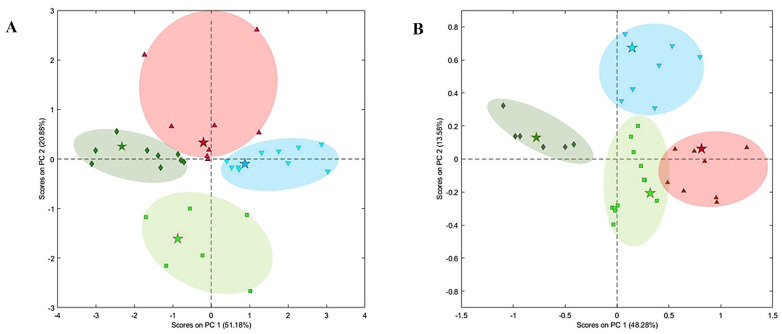
Principal component analysis results of bacteria species: *E. coli* (green square), *B. cereus* (blue inverse triangle), *S. aureus* (dark green diamond), *Salmonella typhimurium* (red inverse triangle). Liquid media (**A**) and solid culture (**B**). Reproduced with permission [40], Copyright 2019, Elsevier.

From a general point of view, Raman spectroscopy uses light scattering phenomenon to acquire information about the molecular vibration patterns of molecular structures. When a substrate is irritated with a laser, two different scattering types (elastic and inelastic) are observed. On the basis of light and molecule interactions, three different types of phenomena can be observed: Rayleigh scattering, anti-Stokes Raman scattering and Stokes Raman scattering [67]. A Raman spectrometer consists of several units: a light source, a monochromator, a sample holder and a detector. Using the Raman spectroscopy technique, it is possible to obtain unique fingerprint properties of molecules on the basis of the related specific functional groups and chemical bonds. Also, using FT-Raman systems, the common fluorescence problem of conventional Raman systems has been overcome. Raman spectroscopy is a strong analytical technique and can be used in a wide variety of applications in the food industry, such as the determination of food ingredients, food additives, food authenticity, raw materials and new compounds [68]. Raman spectroscopy provides various advantages, such as the scanning of the materials through plastic or glass packaging and insensitivity to water molecules [69]. The specificity and unique fingerprinting properties of Raman spectroscopy make the technique a potential alternative for metabolic fingerprinting and metabolic profiling. Until now, various studies have been published for the evaluation of the metabolic detection capabilities of Raman and FT-Raman spectroscopy. Nache et al. (2016) evaluated the capability of Raman spectroscopy combined with chemometrics to predict the metabolic conditions within muscle cells. Their results showed that pH values and pH changes in the meat could be determined on the basis of the recorded Raman spectra [36]. In another study, Chen et al. (2018) used hyperspectral stimulated Raman scattering (hSRS) microscopy and they discovered a new cytoplasmic store of retinoids in *Caenorahbditis elegans.* Also, they reported that they developed a new methodology to track spatiotemporal dynamics in retinoids in living organisms. Stimulated Raman scattering microscopy provided the opportunity to track the retinoid levels and fat storage at the wavelengths of 1580 cm^−1^ and 2857 cm^−1^, respectively. The contribution of the research can be defined as the introduction of a chemical imaging method for the monitoring of retinoids at the subcellular level [70]. Magdas et al. (2019) used FT-Raman spectroscopy for the classification of a wine sample set (*n* = 126) on the basis of the cultivar, geographical origin and vintage. The Raman bands 804 cm^−1^, 985 and 1635 cm^−1^, 960 cm^−1^ were attributed to caffeic acid, caftaric acid and ferulic acid, respectively. The authors stated that caftaric acid was the most powerful marker of production year and wine aging. Their results showed that successful wine discrimination was accomplished on the basis of FT-Raman metabolomics [38]. Jayan et al. (2022) evaluated the capability of surface-enhanced Raman spectroscopy coupled with isotope probing for the analysis of the disinfection of single bacterial cells in chicken carcass wash water. Silver nanoparticles were used to enhance Raman signals of *Escherichia coli* O157:H7. Their results showed that their methodology had the capability to evaluate the metabolic activity of the microorganism *E. coli* on a single-cell level and the authors highlighted the potential of their Raman-based methodology for the evaluation of the metabolic activity of microorganisms in complex matrices such as food products [37]. In a different study, Ma et al. (2021) evaluated the minimum inhibition concentration and AMR profiles of *Campylobacter.* The authors presented the nucleic acid-, protein-, lipid-, peptidoglycan- and unsaturated fatty acid-related Raman bands, which were associated with the amicilin resistance mechanism in a table. According to their report, the Raman spectra revealed the variations in the biochemical properties related to the antibiotic concentration in the case of treatment of the *C. jejuni* isolate with antibiotics [39]. Huayhongthong et al. (2019) evaluated the capability of Raman spectroscopy for the determination of foodborne pathogens. According to their findings, Raman spectroscopy combined with multivariate statistics discriminated the bacterial species *E. coli*, *B. cereus*, *S. aureus* and *S. typhimurium*. The authors stated the potential of Raman spectroscopy with near-IR wavelength excitation for the fast determination of bacteria in food control systems [40]. The authors performed principal component analysis using the spectral range of 500–1600 cm^−1^, which is specific to the characteristic properties of bacteria species. On the basis of PCA results, it was possible to confirm that to principle components of PC1 and PC2 showed significant properties for the discrimination of bacterial species (Figure 2) [40]. As can be seen in Figure 2, different bacterial species (*E. coli*, *B. cereus*, *S. aureus* and *Salmonella typhimurium*) are distinctly clustered according to the principal component analysis of Raman data. 

### 3.3. NIR Applications

#### NIR (Near-Infrared Spectroscopy)

Various natural and agricultural products such as essential oils, seeds, vegetables and fruits need to be explored in terms of their chemical composition. Traditional techniques, such as chromatography and mass spectrometry, have been typically used for the evaluation of the compositional properties of foods and natural products. Near-infrared spectroscopy (NIR) has been widely used for the rapid monitoring of compositional quality properties for the past 40 years [71]. As the main principle, the interaction of light and matter (sample) is measured at the spectral range of 780–2500 nm in near-infrared spectroscopy. The typical NIR bands are composed of O-H, C-H, N-H and S-H bond vibrations of organic and inorganic compounds and the vibrational transitions are related to the overtone and combination bonds [72]. From a general point of view, NIR spectroscopy can be used efficiently for the rapid, precise and low-cost evaluation of the chemical structures of molecules through the absorption of electromagnetic radiation in the NIR spectral range. NIR spectroscopy can be implemented for the quality determination of a wide variety of food products, such as cereals, milk and dairy products, meat, fish, fruit, confectionary, beverages and vegetables. The scientific literature describes various applications of NIR spectroscopy combined with chemometrics for the evaluation of the quality properties of the mentioned food products [73]. In terms of metabolomics, vibrational spectroscopy techniques such as NIR have gained attention since these techniques have the capability of revealing chemical structures on the basis of molecular bonds reflecting the unique fingerprint properties of the analyte. Until now, various NIR spectroscopy studies have been performed to evaluate metabolomics in food matrices.

**Figure 3:**
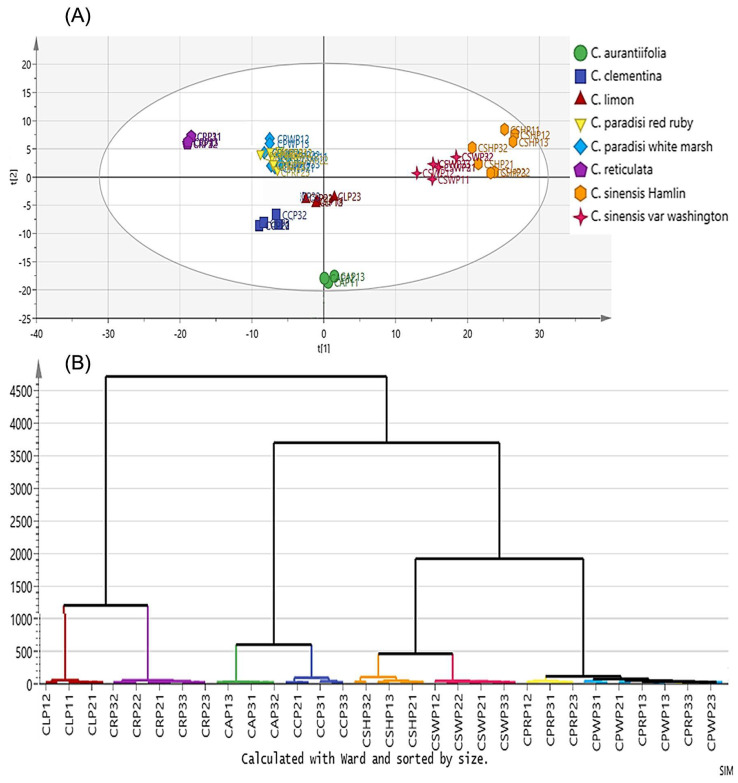
Classification pattern of citrus species: principal component analysis (**A**); hierarchical cluster analysis (**B**). Reproduced with permission [54]. Copyright 2019, Elsevier.

In a previous study, McLeod et al. (2009) used NIR spectroscopy combined with regression models for the prediction of biomass and chemical changes during the beer fermentation process. According to their results, good predictive results were obtained for the prediction of the ethanol concentration, specific gravity, optical density and dry cell weight, and the authors highlighted the importance of the fingerprint region [42]. Cozzolino et al. (2006) combined near-infrared spectroscopy and multivariate analysis for the discrimination of different strains of *Saccharomyces cerevisiae*. Their study showed the considerable potential of NIR spectroscopy combined with chemometrics for the discrimination and identification of different yeast strains. The authors reported VIS and NIR spectroscopy as promising techniques to screen and distinguish yeast strains with deletions in genes that disturb similar metabolic pathways. They used the spectral differences for the classification of yeast species on the basis of their metabolomes and the results were associated with the specific metabolic functions of *Saccharomyces cerevisiae* [44]. Essential oils are high-valued natural products with health-beneficial and aromatic properties. Lafhal et al. (2016) used near-infrared spectroscopy and chemometrics for the quantification of the main compounds in lavender and lavandin. Their results showed that PLS models were capable of predicting linalyl acetate and linalool contents with an error lower than 3% and the main compound-related PLS models showed a favorable R^2^ value ≥0.97. Their results show the considerable potential of NIR spectroscopy and chemometrics for the characterization of lavender and lavandin essential oils. Additionally, the authors stated that the examination of PLS first regression coefficient helped the determination of matabolomic indicators for all lavender/lavandin varieties [45]. In another study, Shawky and Selim (2019) highlighted the applications of NIR spectroscopy in combination with multivariate statistics on the *Citrus* species; they reported the high capability of NIR spectroscopy for the rapid quantification of various bioflavonoids through the fingerprinting capabilities of the technique. Also, they showed the potential of NIR spectroscopy combined with PLS and HCA for the discrimination of different citrus species. As can be understood from Figure 3, a clear classification pattern was obtained using PCA and HCA. According to their findings, PC1 and PC2 explained 52.9% and 20.3% of the results, respectively. C. sinensis Hamlin and C. sinensis var. Washington peel extracts are distinctly classified on the positive side of the PC1 graph when compared to the other species. Additionally, HCA revealed the hidden relationship between different citrus species. The authors correlated the close relationship of the red and white varieties of C. paradise in the HCA dendrogram with their chemical similarities [54]. In a previous contribution, Krause et al. (2015) utilized NIR spectroscopy combined with partial least squares regression for the quality analysis of brewing malt. Their study showed the capability of NIR spectroscopy as a fingerprinting technique for the classification of the processability of malt [56]. They obtained accuracy values higher than 95% both in calibration and validation.

Li et al. (2014) developed a fast, cost-effective, and accurate NIR- and chemometrics-based methodology for the discrimination of Chinese liquors. The authors presented the results of discrimination models built using different wavelengths. The PCA-LDA, SVM and SIMCA models showed % classification ratios of 98.89, 95 and 97.22, respectively, in the spectral range of 570–1848 nm. Also, the spectral range of 1300–1848 nm yielded a classification ratio higher than 94% for all discrimination models [60]. They obtained the best results using PCA-LDA (principal component analysis), with a prediction ability of 98.94%, on a training set that could be used to combat fraudulent samples in the market. In a previous contribution, Li et al. (2007) developed a nondestructive, no-contact methodology for the quantification of the soluble solid content (SSC) of a tea soft drink using vis-NIR spectroscopy. The authors reported that five fingerprint spectra (490, 498, 554, 929 and 970 nm) were detected for the soluble solid content. The wavelength range of the spectroradiometer was selected as 325–1075 nm. The multivariate calibration models of partial least squares (PLS) and multiple linear regression (MLR) were selected to build regression models and a favorable regression coefficient of 0.981 was obtained [61]. Liu et al. (2011) evaluated the potential of visible- and near-infrared (Vis-NIR) spectroscopy for the nondestructive determination of the soluble solid content (SSC) and vitamin C (VC) in Gannan navel oranges. Reasonable and unacceptable PLSR prediction results were obtained for the soluble solid content and vitamin C content, respectively. However, multiple linear regression models yielded better prediction results for te SSC and VC values [62]. Filho et al. (2019) employed portable near-infrared (MicroNIR) spectroscopy for the development of a rapid and reliable phenotyping technique to evaluate cashew apple compositions. The authors stated that MicroNIR spectroscopy could be considered a rapid, nondestructive and cost-effective tool to obtain simultaneous results [66].

## 4. Conclusions

In the last decades, there has been increasing interest in the research of metabolomics using various analytical techniques including targeted and untargeted analyses. Targeted metabolomics analysis techniques are generally used for the evaluation of a set of metabolites. Metabolomic profiling is capable of evaluating a wide variety metabolites and, generally, high-resolution chromatography techniques are used. Metabolomic fingerprinting can be used to obtain the specific fingerprint properties of biological systems, foods, agricultural products and natural products. Vibrational spectroscopy techniques, such as FTIR, NIR and Raman, provide fingerprinting information about the molecules in the composition of investigated compounds. Today, it is of great importance to acquire key information about structural ingredients to ensure the quality and safety of food and agricultural products. In this context, the application of fingerprinting spectroscopy techniques in combination with pattern recognition analyses and multivariate statistics have considerable potential as cutting-edge solutions to a wide variety of quality and safety problems in the food industry. In conclusion, this review illustrated the applications of metabolomic fingerprinting and profiling for the evaluation of various specific properties using high-throughput FTIR, NIR and Raman spectroscopy techniques combined with pattern recognition methodologies, which have been prevalent for last 15 years or so. Existing research shows the high capability of vibrational spectroscopy combined with pattern recognition analysis in determining the authenticity, quality, safety and the other essential parameters of foods on the basis of metabolomic fingerprinting. There is a need for further studies to evaluate the various challenging food safety and authenticity issues present in the food supply chain. In particular, studies presenting developments and innovations in artificial intelligence applications in this field are required as these have considerable potential for the improvement of the scientific and practical knowledge of researchers and the community.

## Figures and Tables

**Table 1:** Overview of reported studies using Fourier transform infrared (FTIR), Raman and NIR spectroscopy to determine authenticity, quality, safety and the other essential parameters in foods.

Detection Technology	Commodity	Type of Food Product	Parameters Measured	Data Acquisition	Data Treatment	References
FTIR	Fruit	Banana	Carbohydrates, proteins, and lipids	4000–500 cm^−1^	PCA	[19]
	Herbal	*Phaleria macrocarpa* (*Mahkota Dewa*)	Functional groups (CHO, -COOH, -NO_2_, -NH, and -OH)	4000–400 cm^−1^	SIMCA	[20]
	Herbal	Some Medicinal Plants (*Glycyrrhiza glabra* root aqueous, *Terminalia chebula* aqueous, *Zingiber officinale*, *Ocimum sanctum* leaf aqueous, *Piper longum*, *Curcuma longa*	Functional groups (such as phenolics (-OH), carbonyl (C=O), aldehyde (CH=O), ether (C-O-C), aromatic (C=C), and alkyl groups –CH)	4000 to 500 cm^−1^	-	[21]
	Herbal	Ginseng leaves	Active ingredients (ginsenosides, polysaccharides, triterpenoids, flavonoids, volatile oils, polyacetylenic alcohols, peptides, amino acids, and fatty acids)	4000 to 400 cm^−1^	PCA, HCA, PLS-DA	[22]
	Cereal	Wheat Genotypes	FTIR-based biomarkers (*Fm482, Fm576, Fm1251, Fm1465, Fm1502*, and *Fm1729*)	4000 to 400 cm^−1^	PCA, LDA	[23]
		Wheat	Mycotoxin deoxynivalenol (DON)	650 and 4000 cm	PLS1, MLR	[24]
	Coffee	Indonesia robusta and arabica coffee	Functional groups (ester/lactone, aldehyde, ketone, aromatic acids, and aliphatic)	4000–400 cm^−1^	PCA, PLS	[25]
	Dairy	Butter	Functional groups (fatty acids, methylene groups, aliphatic groups, CH_3_ groups)	4000–650 cm^−1^	PLS	[26]
	Oil	Corn oil	Aflatoxins 15-acetyldeoxynivalenol	4000–600 cm^−1^	PCA, MSC	[27]
	Bacteria	*Debaryomyces hanseni* (cheese)	Origin of isolation	4000 and 400 cm^−1^	RAPD	[28]
	Fungus	*Phytopathogenic fungus* A. alternata (Greek medicinal and aromatic plants)	Biochemical composition (lipids, proteins and a ratios of lipids/amide II, amide I/total amides and amide II/total amides)	4000–400 cm^−1^	PCA	[29]
	Meat	Minced meat	Temperature of storage, the initial contamination, pH	4000 to 400 cm^−1^	PCA	[30]
	Oil	Pomegranate kernel oil	Quality parameters according to their respective cultivars (refractive index, peroxide value, total phenolic content, refractive index, total carotenoid content)	4000–400 cm^−1^	PCA and OPLS-DA	[31]
		Peanut oil	Aflatoxin B1 (AFB1) and aflatoxin (AFT)	4000 to 650 cm^−1^	MDT	[32]
		Peanut	Aflatoxin B_1_	4000 to 575 cm^−1^	PCA	[33]
	Beverage	Fermented alcoholic beverages	Ethanol	1200–850 cm^−1^	PLS	[34]
FT-RAMAN	Vegetables	Carrot	Carbohydrates, carotenoids, and polyacetylenes	4000 to 100 cm^−1^	PCA, PLS	[35]
RAMAN	Meat	Porcine meat	pH	2105 to 323 cm^−1^	MSC	[36]
	Meat	Chicken carcass	*Escherichia coli* cells	500–3500 cm^−1^	PCA, LDA	[37]
	Beverage	Wine	Ethanol	1000–0 cm^−1^, 3600–0 cm^−1^	SLDA	[38]
	Drug	Antibiotics (*Campylobacter jejuni*)	AMR (antimicrobial resistance) profile	400 to 1800 cm^−1^	HCA, PCA	[39]
	Bacteria strains	foodborne microorganisms (*E. coli* ATCC 25922, *B. cereus* ATCC 11778, *S. aureus* ATCC 13565 and *Salmonella typhimurium* ATCC 13311)	Acids and proteins	500–1600 cm^−1^	PCA	[40]
	Essential oils	Lavender (*Lavandula angustifolia*) and lavandin essential oils	Major terpenoid composition, eucalyptol, camphor, β-Caryophyllene, eucalyptol, linalyl acetate, inalool, β-caryophyllene	90–4000 cm^−1^	PCA, PLS-DA	[41]
NIR	Beverage	Beer	Fermentation parameters (ethanol concentration, specific gravity (SG), optical density, and dry cell weight	10,000 to 4000 cm^−1^	PLS-R	[42]
		Distilled Alcoholic Beverages	Methanol and ethanol	1720–1660 nm		[43]
	Yeast	*Saccharomyces cerevisiae*	O–H second overtone of water and ethanol; C–H_3_ stretch first overtone or with compounds containing C–H aromatic groups	400–2500 nm	LDA, PCA	[44]
	Essential oils	Lavender (*Lavandula angustifolia*) and lavandin essential oils	Linalool and eucalyptol content	4500–9000 cm^−1^	PCA, PLS- DA	[45]
	Cereal	Maize	Aflatoxigenic *Aspergillus* spp. contamination	800–2600 nm	PCA	[46]
		Maize	Ergosterol and fumonisins content	400–2498 nm	PCA	[47]
		Maize	*Aspergillus flavus* fungi	900–2500 nm	PLS-DA	[48]
		Barley	Deoxynivalenol (DON)	10,000 to 4000 cm^−1^	PLS-DA, PLS-R	[49]
		Hulled barley	*Fusarium*	1175 to 2170 nm	PLS-DA	[50]
		Hulled Barley, Naked Barley, and Wheat	*Fusarium*	360–2500 nm	PLS-DA	[51]
		Wheat	Deoxynivalenol (DON)	570–1100 nm	PLS	[52]
		Rice	Aflatoxigenicfungal contamination	950–1650 nm	PLSR	[53]
FT-NIR	Fruit	Citrus species peels	Bioflavonoids (diosmin and hesperidin)	12,000–4000 cm^−1^	HCA, PCA, PLSR	[54]
	Coffee	Green coffee beans	Ochratoxin A (OTA)	800–2500 nm	PLS-DA	[55]
	Cereal	Malt	Lautering	800–2500 nm	PLS-DA, PCA	[56]
		Wheat	Deoxynivalenol (DON)	10,000–4000 cm^−1^	PLS, DA	[57]
		Durum wheat	Deoxynivalenol (DON)	10,000 to 4000 cm^−1^	LDA, PLS	[58]
		Bulk wheat	Deoxynivalenol (DON), moisture content (MC)	10,000 to 4000 cm^−1^	PLS	[59]
Vis/NIR	Beverage	Chinese liquor	Alcohol degree, age, flavor	570–1848 nm	PCA, LDA, SIMCA	[60]
	Beverage	Tea soft drink	Soluble solids content	425–1000 nm	PLS, MLR	[61]
	Fruit	Gannan navel oranges	Soluble solids content and vitamin C	350–1800 nm	PLSR	[62]
	Cereal	Wheat	Toxigenic fungal infection	600 to 1600 nm	PCA, LDA, PLSR	[63]
		Corn	Aflatoxigenic fungus and aflatoxin	400–2500 nm	PLS-DA	[64]
	Nut	Peanut	Aflatoxin B1 (AFB1)	400–2500 nm	PLS-DA	[65]
MicroNIR	Nut	Cashew apple	The °Brix, total acidity, and concentration of ascorbic acid (vitamin C)	1150–2170 nm	PCA, HCA	[66]

## Data Availability

Not applicable.
